# Dissecting Missing Heritability in Rare Inherited Macular Dystrophies

**DOI:** 10.3390/genes17080979

**Published:** 2026-08-20

**Authors:** Deirdre Harford, Marcus Conway, Bridget Moran, Julia Zhu, Jacqueline Turner, Adrian Dockery, James J. O’Byrne, D. Ian Flitcroft, Tomás Burke, Kirk A. J. Stephenson, G. Jane Farrar, David J. Keegan

**Affiliations:** 1 Clinical Ophthalmic Genetics Unit, Mater Misericordiae University Hospital, Eccles Street, D07 R2WY Dublin, Ireland; 2 Next Generation Sequencing Laboratory, Mater Misericordiae University Hospital, Eccles Street, D07 R2WY Dublin, Ireland; 3 Children’s Health Ireland at Temple Street Hospital, D01 XD99 Dublin, Ireland; 4 Smurfit Institute of Genetics, Trinity College Dublin, D02 PN40 Dublin, Ireland

**Keywords:** macular dystrophy, inherited eye disease, missing heritability, ophthalmology, ophthalmic genetics, deep phenotyping

## Abstract

Background/Objectives: To describe the genetic resolution rate, molecular findings, and genotype–phenotype correlations of non-*ABCA4* and non-*BEST1* inherited macular dystrophies (IMDs) within an Irish inherited retinal disease (IRD) registry. Methods: Retrospective review of individuals with a clinical diagnosis of macular or cone dystrophy. Comprehensive phenotyping (dilated ocular biomicroscopy, multimodal retinal imaging, visual electrophysiology) and genetic testing (panel-based next-generation sequencing, single-gene testing, whole exome/genome sequencing, WES/WGS). Variants were interpreted using ACMG AMP criteria, and genotype-phenotype match was confirmed through multidisciplinary review. Results: 232 patients with macular/cone dystrophies were identified. *ABCA4* and *BEST1* accounted for most molecular diagnoses (47.4%). Removing *ABCA4* and *BEST1*, 59.0% of IMDs were genetically unresolved, higher than the rate in general IRD cohorts. Deep phenotyping enabled diagnostic reclassification in 13/72 (18.1%), namely achromatopsia, congenital stationary night blindness, and oculocutaneous albinism. Further genetic testing resolved 35/72 (48.6%) of those unresolved on first-line testing, with *PRPH2* being most prevalent (n = 12), followed by *GUCY2D*, *CRB1*, *PROM1*, and *CRX*. Characteristic phenotypic signatures—such as *CRB1*-associated retinal thickening and retinoschisis or *PROM1*-associated Stargardt-like changes—supported known genotype–phenotype correlations. Conclusions: Genetic resolution rates for rare IMDs remain lower than pan-retinal IRD phenotypes. Beyond *ABCA4* and *BEST1*, IMDs exhibit substantial genetic and phenotypic heterogeneity (24 genotypes in this cohort), with low molecular diagnostic rates despite comprehensive sequencing approaches. Detailed multimodal phenotyping (i.e., structural, functional and extra-ocular) is essential to refine diagnosis, guide genetic testing and interpret candidate variants. Genetic testing is challenging when the retinal phenotype is advanced (i.e., atrophy) or lacks pathognomonic features. Meticulous phenotyping (functional, structural and systemic) and broader genomic strategies (e.g., WES/WGS) may further increase diagnostic yield, though gene panel content is constantly improving. Consistently improving molecular diagnostic rates will ensure equitable access to emerging gene-specific therapies.

## 1. Introduction

Inherited macular dystrophies (IMDs) are a heterogeneous group of inherited retinal diseases (IRDs) characterized by bilateral, grossly symmetrical macular abnormalities and variable loss of central vision [1]. IMDs are the second largest clinical group of IRDs after generalized photoreceptor dystrophies (i.e., retinitis pigmentosa, RP) [2]. While the pathology in IMDs predominantly affects the central retina, both widefield multimodal imaging and visual electrophysiology may demonstrate more widespread retinal dysfunction [e.g., generalized dysfunction of the retinal pigment epithelium (RPE) in Best disease] [3]. Although individually rare, IRDs are now the leading cause of blindness in the working-age population in England, Wales and Australia [4,5]. With the arrival of gene therapy for biallelic *RPE65*-associated IRD, and the plethora of novel therapies for IRDs in clinical trials, ensuring accurate phenotyping and genetic confirmation will improve access for those who may benefit. However, patients with IMDs have a lower rate of genetic resolution than generalized pan-retinal dystrophies, putting them at a further disadvantage (e.g., understanding of prognosis, access to novel disease-modifying therapies). Although the Irish IRD cohort has achieved a positive molecular diagnosis rate of 60–70% on panel-based next-generation sequencing (NGS), this figure is much lower (44%) for those affected by IMDs [6,7].

A primary challenge in IMDs is their broad clinical and genetic heterogeneity. This is especially true for those with advanced disease, where characteristic clinical features seen early in the disease may be masked by progressive chorioretinal atrophy. The most common monogenic macular dystrophies include Stargardt disease (STGD1), Best disease, *PRPH2*-associated maculopathy, X-linked retinoschisis (XLRS), Sorsby fundus dystrophy and pattern dystrophy [3]. However, there are other, less well-characterized macular dystrophies, including phenocopies of the more common disease entities, which frequently go unresolved even though novel gene discovery is ongoing (e.g., *WDR19*, *C19orf44*, *ZZEF1-ALOX15*, *AP5Z1/AP5B1*) [8,9,10,11]. Herein, we describe the molecular resolution rate, genetic findings, and genotype-phenotype correlations of IMDs in an Irish population.

## 2. Materials and Methods

Ethical approval for this study was granted by the Institutional Review Board of the Mater Misericordiae University Hospital and Mater Private Hospital (MMUH IRB1/378/1358), Dublin, Ireland, prior to commencement. All work was carried out in accordance with the Declaration of Helsinki and local data protection regulations (GDPR). All patients provided informed consent for study participation and publication of anonymized data.

A retrospective review of those in the registry with a clinical diagnosis of macular dystrophy (including STGD1, Best disease, *PRPH2*-associated maculopathy, XLRS, Sorsby fundus dystrophy and pattern dystrophy) and cone dystrophy was performed. Patients and relevant family members were recruited and assessed at the Mater Misericordiae University Hospital or Mater Private Hospital (Dublin, Ireland).

Meticulous phenotyping was performed including clinical history, family history, and assessment for syndromic features as previously described [12]. Ophthalmic phenotyping included best corrected visual acuity (BCVA) in logarithm of the minimum angle of resolution (LogMAR) format, applanation tonometry and dilated slit lamp biomicroscopy. Multimodal imaging included ultra-widefield fundus photography and autofluorescence (‘California’, Optos Plc, Dunfermline, UK) and spectral domain optical coherence tomography (OCT; Cirrus 6000; Carl Zeiss Meditec AG, Jena, Germany). Visual field (VF) to the IV4e target (Goldmann Visual Field, Haag-Streit, Switzerland; or Humphrey Visual Field Analyzer; Carl Zeiss Meditec) and International Society for the Clinical Electrophysiology of Vision standard electrophysiology (Diagnosys LLC, Lowell, MA, USA) were documented as available.

Initial genotyping was performed in the affiliated research-grade laboratory (Ocular Genetics Unit, Smurfit Institute of Genetics, Trinity College Dublin, Dublin, Ireland) using target capture NGS assessing a panel of 256 IRD-associated genes as previously described [13]. DNA was isolated from either blood (DNA Blood Maxi Kit, Qiagen, Hilden, Germany) or saliva (Oragene-DNA, DNA Genotek, Kanata, ON, Canada) samples from participants. Sample preparation was carried out using a hybridization-based target capture sequencing method [13]. All genomic locations refer to the Hg38 reference genome (Homo sapiens GRCh38). The American College of Medical Genetics and Genomics and the Association for Molecular Pathology (ACMG-AMP) criteria for classifying pathogenic variants were used to interpret candidate variants with modifications as per ClinGen expert panels (https://clinicalgenome.org/working-groups/clinical-domain/ocular-cdwg/, accessed on 2 June 2026) [14].

Confirmed variants were discussed using a multidisciplinary team (MDT) approach (ophthalmic clinicians, physicians, clinical and molecular geneticists, genetic counsellors) [15]. Published evidence, population databases (e.g., RetNet, ClinVar, gnomAD, LOVD) and the ACMG-AMP criteria were used to confirm genotype-phenotype matches prior to genetic counselling of patients. Patients confirmed to have *ABCA4*- and *BEST1*-associated IMD on first-line genetic testing were excluded from the analyzed cohort of patients. Patients who remained genetically unresolved after initial NGS panel testing underwent phenotypic reassessment and, in suitable cases, were further investigated with single gene, whole exome (WES) or whole genome sequencing (WGS) as appropriate. Prime candidate variants from this assessment were confirmed by clinically accredited laboratories (Manchester Centre for Genomic Medicine, Manchester, UK or Blueprint Genetics, Helsinki, Finland) by resequencing patient DNA to confirm detection of the variant(s) in question. Candidate variants were assessed using the accredited lab’s interpretation pathway (https://www.blueprintgenetics.com/interpretation/, accessed on 7 August 2026).

Due to the rare nature of individual IMD genotypes, this study was not powered for accurate quantitative statistical analysis. However, statistical data are used for illustrative purposes throughout (Microsoft Excel 2024, Dublin, Ireland and SPSS Version 20, IBM, Armonk, NY, USA). Descriptive statistics are reported as mean and standard deviation for numerical data and percent for categorical data.

## 3. Results

### 3.1. Genetic Findings

A clinical diagnosis of IMD was made in 232 patients. Of these, 69.0% (n = 160) were genetically resolved by 13 genotypes on first-line testing, the most common being biallelic *ABCA4* (STGD1, 35.8%, n = 83), heterozygous *BEST1* (autosomal dominant Best disease, 11.6%, n = 27) and hemizygous *RS1* (X-linked retinoschisis, 9.1%, n = 21).

However, 31.0% (n = 72, mean age 47.0 ± 19 years, 38.9% female) remained genetically unresolved after first-round testing. When *ABCA4* and *BEST1* cases were excluded, the proportion of genetically unresolved cases rose to 59.0% (i.e., 72/122), considerably higher than that reported for pan-retinal photoreceptor dystrophies in the Irish IRD population (17.9%).

Second line genetic testing (single gene, WES, WGS) identified the genetic cause of IMD in a further 48.6% (n = 35/72) of cases, while 2.8% (n = 2/72) had a partial genetic diagnosis (i.e., 1 variant detected in AR disease, *ABCA4* & *CNNM4*), and 41.7% (n = 30, 43.3% female) remained unresolved (Table 1, Supplementary Tables S1 and S2). VUS are reported for 5 patients, as these were *in trans* with a pathogenic/likely pathogenic allele (*ABCC6*, *RDH12* & *TYR*) and/or due to a highly consistent phenotype (*CNNM4*). The mean ages of resolved (47.8 ± 19.5 years) and unresolved (46.0 ± 19.5 years) patients were similar. The most prevalent genotypes identified on second-round testing were heterozygous *PRPH2* (n = 12) and heterozygous *GUCY2D* (n = 4), with 19 genotypes represented. Interestingly, second-line genetic testing identified a further case of *BEST1*-associated disease and 3 cases of *ABCA4*-associated STGD1 (2 biallelic, 1 single allele with a consistent phenotype). Only three novel variants were identified (Supplementary Table S1).

### 3.2. Genotype-Phenotype Correlations

**Figure 1:**
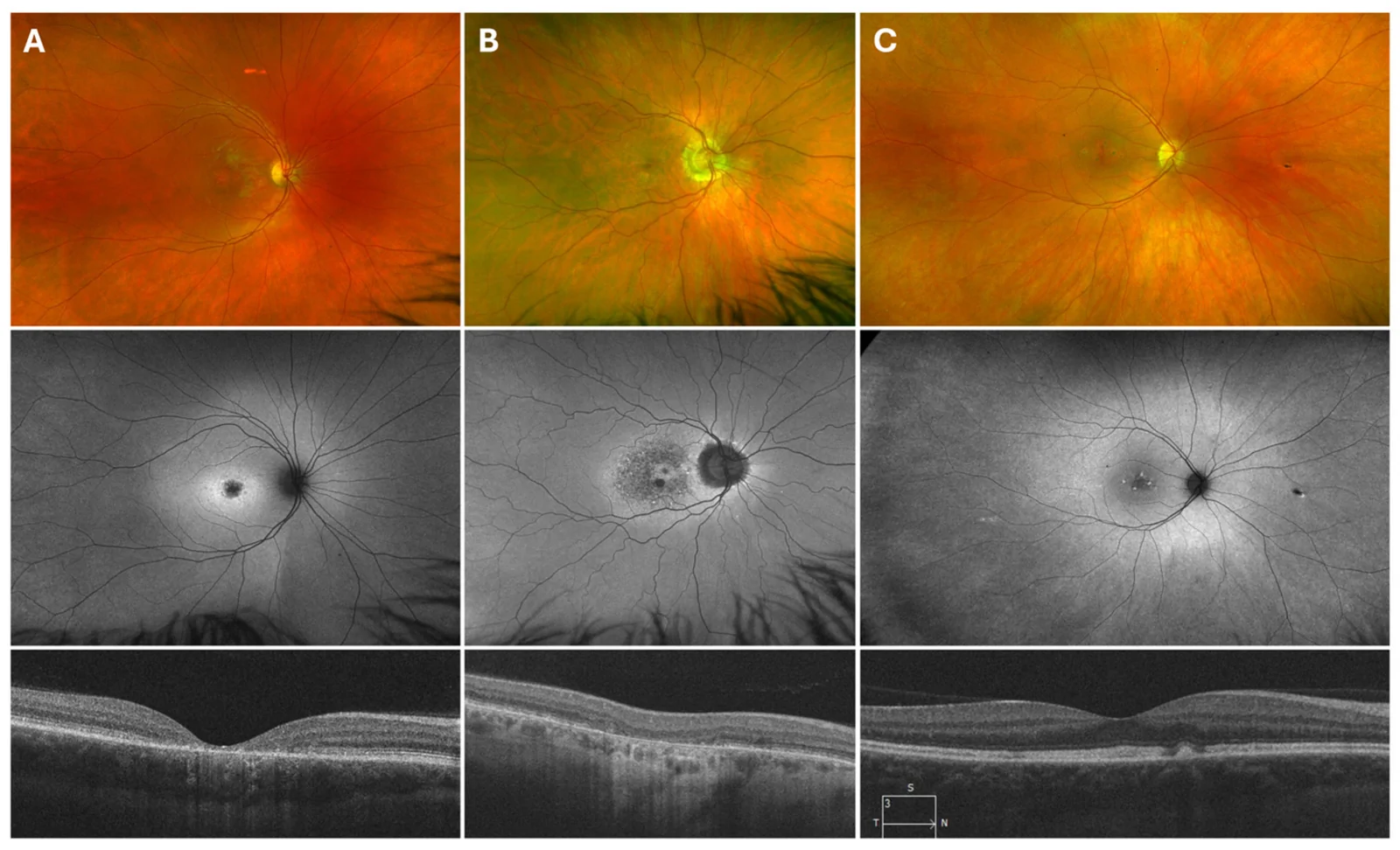
Multimodal imaging of Stargardt-like phenotypes. Top row: Ultra-widefield pseudocolour fundus photographs. Middle row: Ultra-widefield fundus autofluorescence (FAF). Bottom row: spectral-domain optical coherence tomography (SD-OCT) b-scans. (**A**) *ABCA4*-related disease. (**B**) *PROM1*-associated disease. (**C**) *PRPH2*-associated disease. All genotypes share common features, particularly on FAF, namely diffuse background hyperautofluorescence, flecks, and disease restricted to the central macula.

The clinical phenotype of the non-*ABCA4*/*BEST1* cohort often lacked pathognomonic features and demonstrated genetic heterogeneity. Multimodal imaging was available for review in 87.5% (n = 63/72) of cases. ‘STGD-like’ phenotypes were seen in 17/72 (23.6%), of whom only 3 (17.6%) remained unsolved, the remainder being explained by *PRPH2* (n = 7), *ABCA4* (n = 2), *PROM1* (n = 2), *BEST1* (n = 1), *CRB1* (n = 1), and *CRX* (n = 1) (Figure 1A–C).

Seven cases had a bullseye maculopathy phenotype, 4 of whom reached a genetic diagnosis on second-round testing *CRX* (n = 2), *GUCA1A* (n = 2), *ABCA4* (single allele, n = 1) with 3 cases (42.9%) remaining unresolved.

A variable extent of macular atrophy was seen in 25/72 cases (34.7%), of whom 10 (40%) remained genetically unresolved after second-line genetic testing. The resolved cases were explained by *PRPH2* (n = 3), *PROM1* (n = 3), *RP1L1* (n = 1), *GUCY2D* (n = 2), *GUCA1A* (n = 2), *CRX* (n = 1), *CRB1* (n = 2), *CNNM4* (n = 1, single allele), and *C1QTNF5* (n = 1).

Of those who had no candidate variants detected on second-line genetic testing (n = 30), 30.0% had macular atrophy. Six cases (20%) had drusen-like deposits on SD-OCT, only one of whom (16.7%) was genetically resolved (*PRPH2*, Figure 1C).

**Figure 2:**
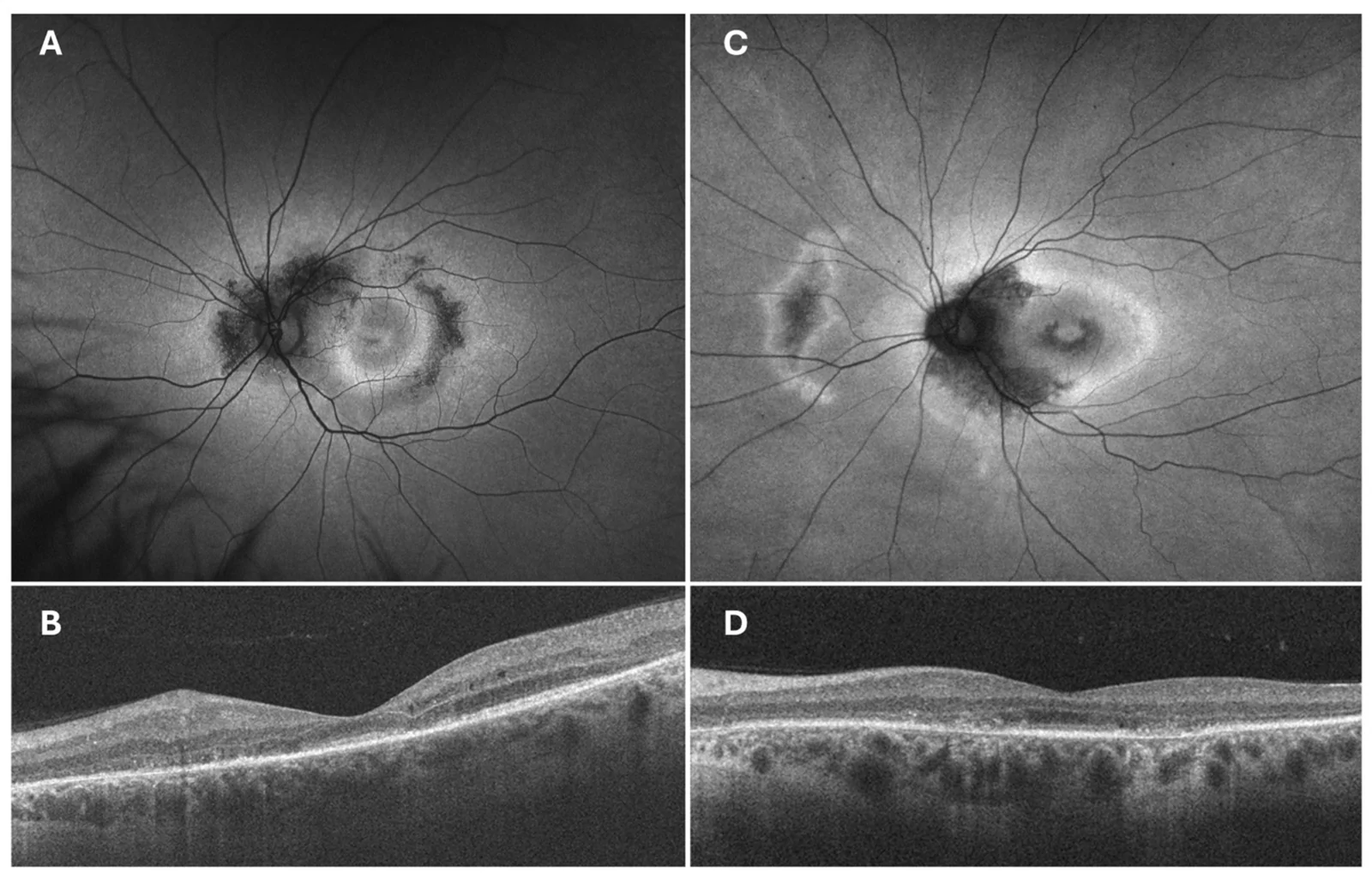
Fundus autofluorescence (**A**,**C**) and SD-OCT (**B**,**D**) images for *CRB1*-associated maculopathy (left column) and *CRX*-associated maculopathy (right column). The FAF phenotype is similar between these 2 genotypes, with predominant macular disease, bullseye maculopathy, and perivascular atrophy, with only the discrete nasal involvement standing out as a unique feature in *CRX*-associated disease (**C**). However, SD-OCT for the *CRB1* case (**B**) shows coarse lamination and a subtle retinoschisis, characteristic of *CRB1*.

Although *CRB1* maculopathy cases typically have characteristic findings of macular retinoschisis and coarse/thickened lamination (Figure 2A), *CRX*-associated maculopathy cases had similar FAF features (Figure 2B), highlighting the limited predictive value of advanced phenotypes in adult patients [16].

Five cases had syndromic features, which aided interpretation of candidate variants [*TYR* albinism, *ABCC6* pseudoxanthoma elasticum (PXE), *CNNM4* Jalili syndrome, *PAX6*]. The *TYR* cases had subtle non-ocular features, but childhood hypopigmentation was elicited on direct questioning and reviewing childhood photographs. This milder OCA1B phenotype was due to compound heterozygous genotypes with the ‘risk factor’ *TYR*: *c.1205G* > *A*, *p.(Arg402Gln)* variant *in trans* with a pathogenic variant (see discussion below).

### 3.3. Phenotypic Heterogeneity

**Figure 3:**
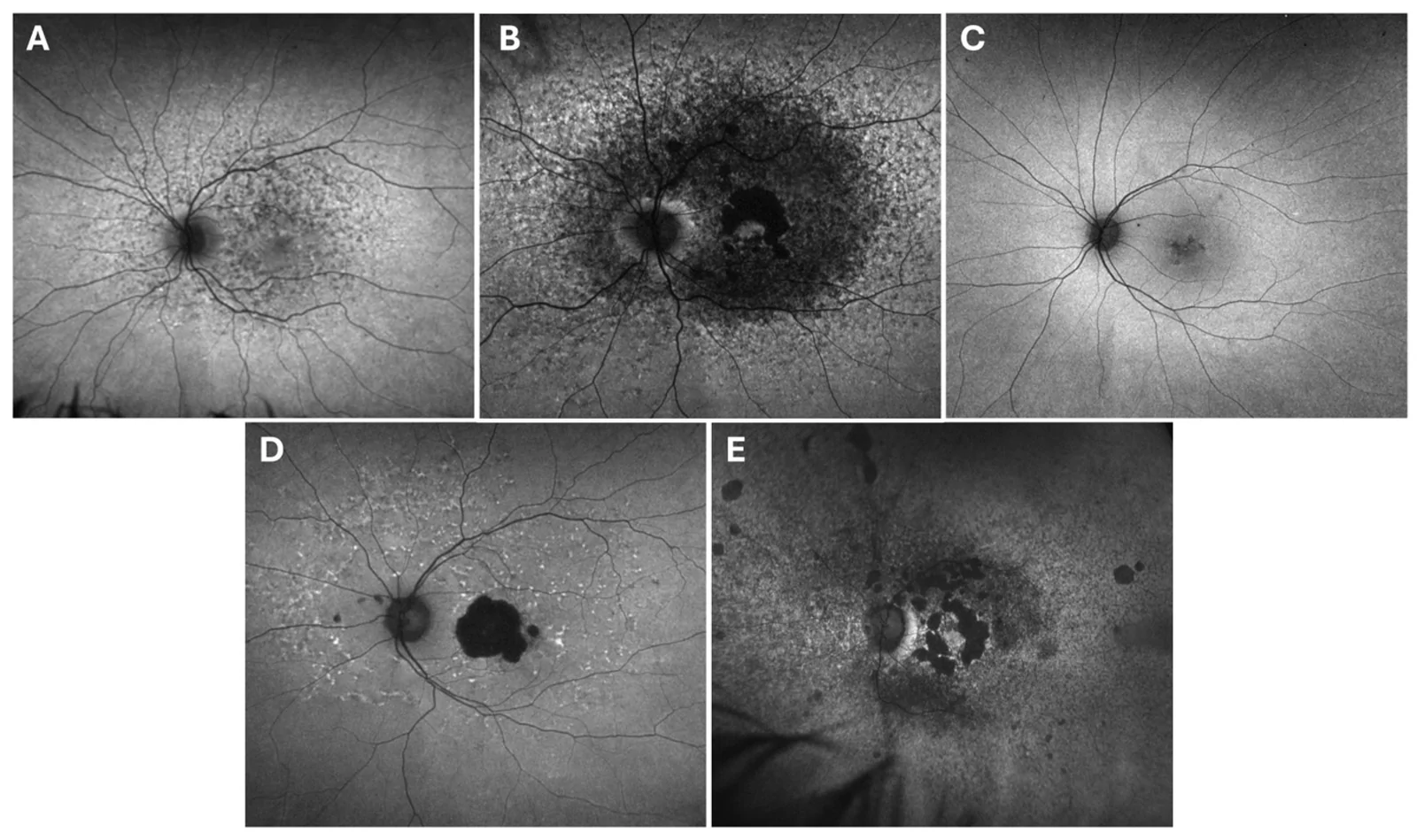
Intrafamilial variability of phenotype associated with the *PRPH2*: c.457A > G, p.Lys153Glu variant. Left eye fundus autofluorescence images of (**A**) 49 year-old female (pedigree 1), (**B**) 67 year-old male (pedigree 2), (**C**) 65 year-old male (pedigree 2), (**D**) 37 year-old female (pedigree 2), and (**E**) 40 year-old male (pedigree 2).

There was marked phenotypic heterogeneity between subjects carrying the same genetic variant, even within families. This was most apparent for the pathogenic *PRPH2*: c.457A > G, p.Lys153Glu variant, which expressed a highly variable retinal dystrophy with prominent macular involvement (Figure 3).

### 3.4. Diagnostic Refinement

On review of phenotype, 13 cases (18.1%) were found to have diagnoses discrete from IMD, namely foveal hypoplasia including albinism (n = 6), congenital stationary night blindness (CSNB, n = 2), normal structure and function (n = 2), optic neuropathy (n = 1), likely solar maculopathy (n = 1), and unilateral idiopathic choroidal neovascularization (n = 1). Re-analysis of the phenotype identified nystagmus in eight cases, suggesting congenital onset disease and thus significantly narrowing candidate genotypes. ERG helped to further guide interpretation of candidate genetic variants based on predominant rod dysfunction (*CACNA1F* incomplete CSNB), cone system dysfunction (*CNGB3*-, *PDE6H*-achromatopsia, ACHM) or normal full-field ERG (*TYR* oculocutaneous albinism, *PAX6* foveal hypoplasia).

## 4. Discussion

We report the clinical and genetic features of IMDs in a cohort with initially negative first-round panel-based NGS. After further genetic testing, the total genetic resolution rate increased from 69.0% to 84.1% (195/232) with 24 distinct genotypes represented. *ABCA4* (36.6%, n = 85/232), *BEST1* (12.1%, 28/232), *RS1* (9.1%, n = 21) and *PRPH2* (6.0%, n = 14/232) were the most prevalent genotypes in the overall cohort.

Though the genetic resolution rate of IMDs was initially less than other IRDs, meticulous phenotyping (as per our “gene-negative” algorithm [17]), including extra-retinal features (e.g., refractive error, axial length, nystagmus) and non-ocular/syndromic features, was instrumental in guiding the next genetic testing steps and in interpreting candidate genetic variants. This highlights the diagnostic shortfall of relying exclusively on the retinal phenotype outside of the three most common IMD genotypes (i.e., *ABCA4*, *BEST1*, *RS1*). Negative genetic findings were most common in cases with non-specific findings (e.g., drusen, 83.3%), significant macular atrophy (40.0%) or bullseye maculopathy (42.9%), but were low for STGD-like phenotypes (17.6%). Patients presenting in adulthood often have some degree of macular atrophy at baseline, which represents a final common pathway of photoreceptor/RPE degeneration. This often obliterates pathognomonic clinical features (e.g., external limiting membrane thickening in *ABCA4* or vitelliform material in *BEST1*), emphasizing the need for earlier phenotyping where possible (e.g., cascade testing within affected pedigrees).

Without a confirmed genotype, patients will be unable to access gene- or variant-specific therapies. Thus, pursuing further genetic analysis is in the best interest of patients with suspected IMD/IRD. Our findings highlight that the precision of phenotyping is the key to identifying the genetic cause of the IMD. The use of the standardized Human Phenotype Ontology (HPO) [https://hpo.jax.org/, accessed on 7 August 2026] terms is now encouraged to assist this process. More in-depth molecular analyses such as WES, WGS, or single-gene sequencing are useful tools, but if the incorrect panel is used for initial testing, a negative result is more likely. While some congenital retinopathies are well-represented on broad IRD panels (e.g., CSNB, achromatopsia), foveal hypoplasia and oculocutaneous albinism are under-represented. For example, Blueprint Genetics’ retinal dystrophy panel tests only 5 of the 26 genes covered on its albinism panel, in addition to *FRMD7* for isolated foveal hypoplasia. Thus, multimodal data from functional (e.g., ERG, nystagmus), structural (e.g., retinal imaging), and non-ocular (e.g., skin & hair pigmentation in OCA) domains give the most accurate indication of the phenotype and guide the most appropriate genetic testing approach (i.e., test type or panel choice) to maximize diagnostic yield. Reanalysis of the phenotype and further genetic analysis in the current cohort led to reclassification of diagnosis in 18.1% of cases.

IMDs may be misdiagnosed as age-related macular degeneration (AMD), particularly in those who present in adulthood [18]. Though there are risk alleles for AMD (e.g., *CFH*, *ARMS2*), it is not a strictly Mendelian condition. A recent publication by the International Age-Related Macular Degeneration Genomics Consortium confirmed 52 risk alleles across 28 loci [19]. For example, deleterious variants in the factor H-like protein 1 (*FHL-1*) gene may cause early accumulation of Bruch’s membrane deposits [20]. The added complication in discriminating AMD from IMDs is the impact of known environmental factors, such as smoking and obesity, again highlighting the importance of non-ocular features [19]. Our group has previously demonstrated the impact of micro-RNAs affecting multiple physiological pathways in patients with AMD [21]. Some patients labelled with a diagnosis of AMD, particularly those with a strong family history, may well have an IMD but have not been referred for assessment in an ophthalmic genetics clinic. As genetic sequencing continues to reduce in cost and increase in availability, this may be appropriate for patients with “dystrophic” or “early-onset” AMD.

The two patients in whom candidate *TYR* variants were detected posed a clinical challenge. Both patients carried the *TYR*: c.1205G > A, p.(Arg402Gln) “risk factor” variant *in trans* with a pathogenic *TYR* allele, namely c.613C > A, p.(Pro205Thr) and c.1111A > T, p.(Asn371Tyr), respectively. Though *TYR*: c.1205G > A is common in the white population (approximately 25% carrier rate), when *in trans* with a different disease-associated *TYR* allele, a milder systemic phenotype may manifest. Though hair and skin are typically hypopigmented or non-pigmented in infancy, cutaneous/hair pigment is often gained with time, and the ophthalmic phenotype may be similarly attenuated [22]. Thus, the systemic phenotype of a patient with hair/skin pigmentation within the normal range for ethnicity, who also has mild or no nystagmus and clinically subtle macular changes (e.g., grade 1–2 foveal hypoplasia) could understandably be missed, and the ophthalmic diagnosis be labelled as ‘macular dystrophy.’ Detection of this hypomorphic *TYR* allele in a compound heterozygous state not only genetically resolves these cases, but confers a favorable, non-progressive visual prognosis for these patients.

Most clinical IRD services globally use first-line NGS gene panels or WES. However, nearly 10% of IRDs are caused by intronic variants (e.g., splice-altering, promoter regions, deep intronic variants, pseudoexons, copy number variation, etc.). Novel intronic variants are unlikely to be detected with this sequencing approach. Genomics England has initiated first-line WGS [23], enabling investigation of both exons and introns. While this may increase the rate of genetic diagnosis, there is a bioinformatics bottleneck as the introns contain huge amounts of data which may deviate significantly from the reference genome. To avoid this issue, the use of “virtual panels” can target IMD/IRD-relevant genes including the intronic regions of these genes.

The lack of or loss of (i.e., atrophy in adulthood) pathognomonic retinal features can delay or hinder accurate diagnosis. The most commonly occurring genetic diagnosis within our cohort on second-line genetic testing was *PRPH2* (12 cases from 5 pedigrees). *PRPH2* encodes the transmembrane glycoprotein peripherin-2, which contributes to the regulation of photoreceptor outer segments [24,25]. *PRPH2*-associated phenotypes are diverse [e.g., vitelliform, Stargardt-like, central areolar choroidal dystrophy (CACD), RP, Leber congenital amaurosis (LCA)]; therefore, genotype-phenotype correlation is poor. However, *PRPH2* should be considered in a wide array of clinical presentations [24,25,26,27,28,29]. Daftarian et al. reported a complex family with wide intrafamilial phenotypic variability including macular chorioretinal atrophy with flecks, CACD, pattern dystrophy, and LCA (homozygous case), all due to the pathogenic *PRPH2*: c.715C > T, p.Gln239* variant [24]. Similarly, in our cohort, 8 patients from 2 pedigrees carried the *PRPH2*: c.457A > G, p.Lys153Glu pathogenic variant with vastly differing severity of retinopathy, though macular involvement was a consistent feature (Figure 3). One case from this pedigree is also described in a recent case report by our group (under review) [29].

Three patients in the current cohort were found to carry deleterious, heterozygous variants in *PROM1*, a gene associated with STGD-like retinopathy, cone-rod dystrophy (CRD) and rod-cone dystrophy [30]. Akin to *PRPH2*, there is often considerable phenotypic heterogeneity even among affected relatives, suggesting that other genetic, epigenetic, or environmental factors may influence expression [30,31,32].

*GUCY2D* variants were identified in 4 cases in the current study. *GUCY2D* encodes a retina-specific guanylate cyclase, which is predominantly expressed in photoreceptor outer segments (cones > rods) [33,34,35]. *GUCY2D* is also associated with diverse retinopathy phenotypes including severe LCA, cone-rod dystrophy, and isolated maculopathy of variable severity (foveal hypoplasia to geographic atrophy) [36]. Variants in *CRB1* may also cause a broad range of retinopathy phenotypes (LCA, RP, maculopathy), though pathognomonic features have been reported (e.g., perivascular sparing of RPE, ‘coarse’ retinal lamination, macular retinoschisis) [37,38,39,40]. Maculopathy is reportedly the most common posterior segment finding in *CRB1*-IRD, with 65% in one study having ill-defined lamination and 24% having disorganization of retinal layers [40]. Awareness of these features and closer investigation of high-resolution OCT may help to identify likely *CRB1* cases. However, these were difficult to elicit in the three adult cases in this cohort (mean age 50.7 years) due to more advanced retinal degeneration.

The Blueprint retinal dystrophy panel used at the time of analysis for this cohort included 266 genes. This has now been expanded to 351 IRD-associated genes, including the mitochondrial genome and recurrent intronic and structural variants, further enhancing the likelihood of molecular diagnosis with current NGS IRD panels. However, supplementary WES/WGS may still be required in certain cases. The development of multi-national registries [https://redgistry.eu/, accessed on 7 August 2026] will enhance our understanding of phenotypic variation with the possibility of large reference phenotype/genotype image banks to aid resolution of cases. The growing use of artificial intelligence (AI) assisted diagnostics may further accelerate this diagnostic process [41].

This study underlines the genetic heterogeneity of IMDs. While *ABCA4* is the most common single IRD/IMD genotype, *ABCA4* and *BEST1* together only represented 48.7% (n = 113/232) of IMDs in this cohort. Variants in 22 other genes underlie 36.2% (n = 84/232) of IMDs in this cohort, with only 12.9% (n = 30/232) remaining genetically unresolved or without any relevant candidate variants. This unresolved population is shrinking with the recent discovery of several novel candidate genes for IMDs (e.g., *WDR19*, *C19orf44*, *ZZEF1*-*ALOX15*, *AP5Z1*/*AP5B1*) [9,10,11].

There are several limitations to this study, including its retrospective design, which increases the risk of selection bias. NGS testing was performed at multiple laboratories and used older panel designs. Were these patients tested today with modern NGS panels with significantly improved gene coverage, a higher rate of genetic diagnosis would be expected on first-line testing. Variant interpretation was performed using the pathways of the accredited testing laboratories. Updated gene-specific ClinGen guidelines are now in effect or being developed for several IRD-associated genes (e.g., *ABCA4*, *GUCY2D*, *CACNA1F*, *PRPH2*) [https://www.clinicalgenome.org/, accessed 12 August 2026].

The genetic diversity and variable expressivity of IMDs/IRDs pose a significant challenge for the development of gene-specific therapies. While clinical trials are ongoing for the most prevalent genotypes, including *ABCA4*-, *BEST1*- and *RS1*-associated IMDs, the rarer genotypes may experience substantial delay in development and approval of therapy, though there is hope that ‘gene agnostic’ strategies may have broad applications in IMDs. For these rare IMDs, confirmation of the genetic cause of disease has prognostic benefit. For example, genetic confirmation of an ACHM, CSNB or OCA diagnosis generally confers a more stationary prognosis when compared with progressive photoreceptor/RPE dystrophies. Additional benefits of a confirmed genotype may include surveillance for potentially life-threatening systemic disease (e.g., *ABCC6*–PXE, *PAX6*–aniridia/Wilms tumor).

## 5. Conclusions

In summary, careful phenotyping, including greater use of HPO terms and appropriate second-line genetic test selection, can maximize the resolution rate for patients with a clinical diagnosis of IMD. This may have prognostic, therapeutic, or systemic surveillance benefits. The non-specificity of clinical features, particularly in older individuals, and potential inclusion of parallel disorders (e.g., ACHM, CSNB, OCA, AMD) may impede genetic diagnosis. The diversity of the genetic alterations underlying IMDs will likely be disadvantageous for developing effective therapies, particularly for rare genotypes and unresolved cases.

Our findings support an enhanced clinically-guided genomic strategy, personalized to each case/pedigree. Though the yield from panel-based NGS is high for pan-retinal IRDs, this is lower in IMDs for the reasons discussed above. Considering the diversity of IMD presentations, the ideal route to an accurate diagnosis is personalized genetic testing with interpretation of candidate variants in the context of the multimodal clinical phenotype including both ophthalmic and non-ocular features.

## Figures and Tables

**Table 1:** Outcome of second-line genetic testing.

Gene	*n* = (%)	Mean Age (y)	Age Range (y)
Unresolved	30 (41.7)	46	9–79
*PRPH2*	12 (16.6)	60	37–76
*GUCY2D*	4 (5.5)	44	28–64
*ABCA4*	3 (4.2)	25	15–40
*CRB1*	3 (4.2)	50	42–58
*PROM1*	3 (4.2)	55	50–63
*CRX*	2 (2.8)	56.5	50–63
*TYR*	2 (2.8)	36	13–59
*ABCC6*	1 (1.4)	58	58
*BEST1*	1 (1.4)	70	70
*C1QTNF5*	1 (1.4)	67	67
*CACNA1F*	1 (1.4)	17	17
*CERKL*	1 (1.4)	22	22
*CNGB3*	1 (1.4)	42	42
*CNNM4*	1 (1.4)	67	67
*GUCA1A*	1 (1.4)	52	52
*KCNV2*	1 (1.4)	19	19
*PAX6*	1 (1.4)	20	20
*PDE6H*	1 (1.4)	29	29
*RDH12*	1 (1.4)	11	11
*RP1L1*	1 (1.4)	55	55

## Data Availability

Source data are available on reasonable request.

## Supplementary Materials

The following supporting information can be downloaded at: https://www.mdpi.com/article/10.3390/genes17080979/s1, Table S1: Genetic findings and ACMG scores; Table S2: Phase of detected variants for patients with relevant genetic test results.
