# Supplementary material for: Dissecting Missing Heritability in Rare Inherited Macular Dystrophies
Source: Genes (Basel). 2026 Aug 20;17(8):979. doi: 10.3390/genes17080979 (PMC13512636; doi:10.3390/genes17080979)
Supplement: Supplementary file 1 [file genes-17-00979-s001.zip › genes-4469287-table S1.pdf]

Supplementary Table S1. Genetic findings and ACMG scores.

Gene (Transcript)	Variant	ClinVar ID	n=	ACMG Class (Points)	REVEL Score (Missense Only)	Criteria
Unresolved	NA	NA	NA	NA	NA	NA
ABCA4 (NM_000350.3)	c.6416G>C, p.(Arg2139Pro)	298222	1	P (16)	Deleterious (Strong) (1)	PM3_VeryStrong, PM1, PP2, PM2, PM5, PP3
	c.5917del, p.(Val1973*)	99419	2	P (12)	NA	PS4_Moderate, PVS1, PM2
	c.4139C>T, p.(Pro1380Leu)	7904	1	P (13)	Deleterious (Moderate) (0.87)	PM3, PP1, PS3, PM1, PP2, PM2, PP3
	c.161G>A, p.(Cys54Tyr)	99065	1	P (11)	Deleterious (Strong) (0.98)	PM3, PP1, PM1, PP2, PM2, PM5, PP3
ABCC6 (NM_001171.5)	c.4016G>A, p.(Arg1339His)	265021	1	LP (8)	Deleterious (Moderate) (0.83)	PM2, PM5, PM1, PP3, PP4
	c.1199C>T, p.(Ser400Phe)	433242	1	VUS (4)	Deleterious (Supporting) (0.67)	PM2, PP3, PP4
BEST1 (NM_004183.4)	c.73C>T, p.(Arg25Trp)	99751	1	P (12)	Deleterious (Moderate) (0.83)	PS4, PM1, PP2, PM2, PM5, PP3
C1QTNF5 (NM_015645.4)	c.489C>G, p.(Ser163Arg)	2126	1	P (18)	Deleterious (Supporting) (0.73)	PS4, PP1, PS3, PS1, PM2, PP3, PM1
CACNA1F (NM_005183.4)	c.245G>A, p.(Arg82Gln)	988786	1	LP (8)	Deleterious (Moderate) (0.81)	PM3_Strong, PM2, PP3_Moderate
CERKL (NM_201548.5)	c.769C>T, p.(Arg257*)	2364	2	P	NA	PM3, PP1, PVS1, PM2
CNGB3 (NM_019098.4)	c.1148del, p.(Thr383Ilefs*13)	5225		P (10)	NA	PVS1, PM2
CNNM4 (NM_020184.4)	c.1660G>T, p.(Ala554Ser)	#	1	VUS (3)	Uncertain (0.36)	PM2, PP4
CRB1 (NM_201253.2)	c.3991C>T, p.(Arg1331Cys)	865928	2	LP (7)	Deleterious (Moderate) (0.87)	PM2, PP3, PM5, PP2, PP4
	c.4142C>T, p.(Pro1381Leu)	973928	2	P (10)	Deleterious (Supporting) (0.7)	PP1, PM1, PP2, PM2, PM5, PP3, PP4
	c.2639A>G, p.(Asn880Ser)	43807	2	LP (7)		PM3, PM2, PM5, PP2
CRX (NM_000554.4)	c.605delG, p.(Cys202Serfs*17)	236495	2	P (14)	NA	PS4, PVS1, PM2

GUCA1A (NM_000409.4)	c.250C>T, p.(Leu84Phe)	225232	1	P (14)	Uncertain (0.49)	PS4, PP1, PS3, PM1, PP2, PM2
GUCY2D (NM_000180.3)	c.2480A>C, p.(Tyr827Ser)	3249829	1	P (12)	Deleterious (Moderate) (0.82)	PS4, PM2, PM1, PM5, PP3, PP4
	c.2513G>C, p.(Arg838Pro)	811743	3	P (16)	Deleterious (Moderate) (0.87)	PS1, PP3, PS2, PM2, PM5, PM1, PP3
KCNV2 (NM_133497.4)	c.(1356+1_1357-1)_(*1_?)del	Not listed	1	P (10)	NA	PVS1, PM2
	c.1637A>C, p.(*546Serext*60)	1421350	1	LP (7)	NA	PP2, PM4, PM2, PM3
PAX6 (NM_001368894.2)	c.109G>C, p.(Ala37Pro)	2954102	1	LP (6)	Deleterious (Moderate) (0.92)	PM1, PP2, PM2, PP3
PDE6H (NM_006205.3)	c.199G>T, p.(Glu67*)	#	2	P (10)	NA	PVS1, PM2
PROM1 (NM_006017.3)	c.1354dupT, p.(Tyr452fs)	372711	2	P (14)	NA	PS4, PVS1, PM2
	c.1142-1G>A	809626	1	P (14)	NA	PS4, PVS1, PM2
	c.303+1G>T	954294	1	P (14)	NA	PS4, PVS1, PM2
PRPH2 (NM_000322.4)	c.457A>G, p.(Lys153Glu)	1175280	8	P (13)	Deleterious (Moderate) (0.86)	PS4, PM1, PP2, PM2, PM5, PP1, PP3
	c.441delT, p.(Gly148Alafs*5)	98668	2	P (14)	NA	PS4, PVS1, PM2
	c.638G>A, p.(Cys213Tyr)	98692	1	P (13)	Deleterious (Strong) (0.95)	PS4, PP1, PM1, PP2, PM2, PM5, PP3,
	c.903_906del, p.(Ser301Argfs*22)	866239	1	P (14)	NA	PS4, PVS1, PM2,
RDH12 (NM_152443.3)	c.184C>T, p.(Arg62*)	2050	1	P (12)	NA	PM3, PVS1, PM2
	c.343+5G>A	#	1	LP (7)	NA	PM2, PM3, PM4, PP3
RP1L1 (NM_178857.5)	c.7203G>T, p.(Ter2401Tyrext*5)	#	1	VUS (5)	NA	PM2, PM4, PP4
TYR (NM_000372.5)	c.613C>A, p.(Pro205Thr)	99572	1	P (10)	Deleterious (Moderate) (0.92)	PM3, PM1, PP2, PM2, PM5, PP3
	c.1205G>A, p.(Arg402Gln)	3779	2	RF	Deleterious (Supporting) (0.7)	PM5, PP3, PP2, PP5, BA1, BS2, BP6
	c.1111A>T, p.(Asn371Tyr)	99534	1	P (14)	Deleterious (Strong) (0.98)	PM3, PS3, PM1, PP2, PM2, PM5, PP3

LP – likely pathogenic. N= number of alleles in the study population. NA – not applicable. P – pathogenic. RF – risk factor. VUS – variant of uncertain significance. #Indicates novel variant.
