# Supplementary material for: Dissecting Missing Heritability in Rare Inherited Macular Dystrophies
Source: Genes (Basel). 2026 Aug 20;17(8):979. doi: 10.3390/genes17080979 (PMC13512636; doi:10.3390/genes17080979)
Supplement: Supplementary file 1 [file genes-17-00979-s001.zip › genes-4469287-table S2.pdf]

Supplementary Table S2. Phase of detected variants for patients with relevant genetic test results.

Gene	Variant 1	Variant 2
ABCA4	c.4139C>T, p.Pro1380Leu	c.161G>A, p.Cys54Tyr
ABCA4	c.6416G>C, p.(Arg2139Pro)	1 allele only
ABCA4	c.5917del, p.(Val1973*)	c.5917del, p.(Val1973*)
ABCC6	c.4016G>A, p.(Arg1339His)	c.1258C>G, p.(Leu420Val)
BEST1	c.73C>T, p.(Arg25Trp)	NA
C1QTNF5	c.489C>G, p.Ser163Arg	NA
CACNA1F	c.245G>A, p.(Arg82Gln)	NA
CERKL	c.847C>T, p.Arg283*	c.847C>T, p.Arg283*
CNGB3	c.1148del, p.(Thr383Ilefs*13)	c.1148del, p.(Thr383Ilefs*13)
CNNM4	c.1660G>T, p.Ala554Ser	1 allele only
CRB1	c.3991C>T, p.Arg1331Cys	c.4142C>T, p.Pro1381Leu
CRB1	c.3991C>T, p.Arg1331Cys	c.4142C>T, p.Pro1381Leu
CRB1	c.2432A>G, p.Asn811Ser	c.2432A>G, p.Asn811Ser
CRX	c.605delG, p.Cys202SERfs*17	NA
CRX	c.605delG, p.Cys202SERfs*17	NA
GUCA1A	c.250C>T, p.Leu84Phe	NA
GUCY2D	c.2480A>C, p.Tyr827Ser	NA
GUCY2D	c.2513G>C, p.(Arg838Pro)	NA
GUCY2D	c.2513G>C, p.(Arg838Pro)	NA
GUCY2D	c.2513G>C, .(Arg838Pro)	NA
KCNV2	c.(1356+1_1357-1)_(*1_?)del	c.1637A>C, p.(*546Serext*60)

PAX6	c.109G>C, p.(Ala37Pro)	NA
PDE6H	c.199G>T, p.Glu67*	c.199G>T, p.Glu67*
PROM1	c.1354dupT, p.Tyr452fs	NA
PROM1	c.1354dupT, p.Tyr452Leufs*113	c.1142-1G>A
PROM1	c.303+1G>T	NA
PRPH2	c.457A>G, p.Lys153Glu	NA
PRPH2	c.441delT, p.Gly148Alafs*5	NA
PRPH2	c.638G>A, p.Cys213Tyr	NA
PRPH2	c.457A>G, p.Lys153Glu	NA
PRPH2	c.457A>G, p.Lys153Glu	NA
PRPH2	c.457A>G, p.Lys153Glu	NA
PRPH2	c.457A>G, p.Lys153Glu	NA
PRPH2	c.457A>G, p.Lys153Glu	NA
PRPH2	c.457A>G, p.(Lys153Glu)	NA
PRPH2	c.441delT, p.Gly148Alafs*5	NA
PRPH2	c.457A>G, p.Lys153Glu	NA
PRPH2	c.903_906del, p.(Ser301Argfs*22)	NA
RDH12	c.184C>T, p.(Arg62*)	c.343+5G>A
RP1L1	c.7203G>T, p.Ter2401Tyrext*5	NA
TYR	c.613C>A, p.(Pro205Thr)	c.1205G>A, p.(Arg402Gln)
TYR	c.1111A>T, p.(Asn371Tyr)	c.1205G>A, p.(Arg402Gln)

NA – not applicable (phenotype is associated with either dominant or X-linked disease and requires no second variant to be expressed).
